# Comprehensive Analysis and Expression Profiling of the *OsLAX* and *OsABCB* Auxin Transporter Gene Families in Rice (*Oryza sativa*) under Phytohormone Stimuli and Abiotic Stresses

**DOI:** 10.3389/fpls.2016.00593

**Published:** 2016-05-03

**Authors:** Chenglin Chai, Prasanta K. Subudhi

**Affiliations:** School of Plant, Environmental, and Soil Sciences, Louisiana State University Agricultural CenterBaton Rouge, LA, USA

**Keywords:** drought, salinity, ABA, auxin transport, gene expression

## Abstract

The plant hormone auxin regulates many aspects of plant growth and developmental processes. Auxin gradient is formed in plant as a result of polar auxin transportation by three types of auxin transporters such as *OsLAX, OsPIN*, and *OsABCB*. We report here the analysis of two rice auxin transporter gene families, *OsLAX* and *OsABCB*, using bioinformatics tools, publicly accessible microarray data, and quantitative RT-PCR. There are 5 putative *OsLAXs* and 22 putative *OsABCBs* in rice genome, which were mapped on 8 chromosomes. The exon-intron structure of *OsLAX* genes and properties of deduced proteins were relatively conserved within grass family, while that of *OsABCB* genes varied greatly. Both constitutive and organ/tissue specific expression patterns were observed in *OsLAXs* and *OsABCBs*. Analysis of evolutionarily closely related “gene pairs” together with organ/tissue specific expression revealed possible “function gaining” and “function losing” events during rice evolution. Most *OsLAX* and *OsABCB* genes were regulated by drought and salt stress, as well as hormonal stimuli [auxin and Abscisic Acid (ABA)], which suggests extensive crosstalk between abiotic stresses and hormone signaling pathways. The existence of large number of auxin and stress related *cis*-regulatory elements in promoter regions might account for their massive responsiveness of these genes to these environmental stimuli, indicating complexity of regulatory networks involved in various developmental and physiological processes. The comprehensive analysis of *OsLAX* and *OsABCB* auxin transporter genes in this study would be helpful for understanding the biological significance of these gene families in hormone signaling and adaptation of rice plants to unfavorable environments.

## Introduction

Auxin is an important hormone for plants' growth, development, and responses to environmental stresses (Ghanashyam and Jain, 2009; Gallavotti, 2013; Rahman, 2013; Darling-Novak et al., 2014). Auxin biosynthetic pathway in plants is highly conserved (Zhao, 2014) and auxin is mainly synthesized in leaf primordium, young leaves, and developing seeds (Ljung et al., 2002). From the sites of its biosynthesis, auxin is redistributed throughout the whole plant where it plays important role in a variety of developmental and adaptive processes via two distinct and indispensable pathways: long-distance transportation in a fast, non-polar fashion through phloem, and short-distance transportation in a slow, cell-to-cell, and polar manner (Swarup and Bennett, 2003; Michniewicz et al., 2007). Polar auxin transportation (PAT), which leads to an auxin gradient in the targeting tissues, is essential for many auxin-dependent developmental processes such as embryonic development, organ formation and positioning, vascular tissue development, root meristem maintenance, root and stem tropisms, and apical dominance (Michniewicz et al., 2007; Swarup and Péret, 2012; Balzan et al., 2014). Three auxin transporter gene families are primarily responsible for PAT, including the AUXIN1 (AUX1)/LIKE AUX1 (LAX) influx carrier family, the PIN-FORMED (PIN)/PIN-like efflux carrier family, and the POLYGLYCOPROTEIN (PGP) /MULTIDRUG RESISTANCE (MDR)/ATP-binding cassette transporters of the B class (ABCB) efflux/conditional transporter gene family (Geisler et al., 2005; Terasaka et al., 2005; Forestan et al., 2010; Barbez et al., 2012; Swarup and Péret, 2012).

AUX1/LAX auxin influx carrier proteins were first identified in Arabidopsis, which belong to the amino acid permease family of proton-driven transporters and facilitate entry of auxin, indole-3-acetic acid (IAA), into cells (Swarup et al., 2004). Despite high conservation in protein sequence and biochemical functions, the Arabidopsis *AUX1/LAX* auxin influx carrier genes generally exhibit non-redundant expression pattern and play a distinct role in many auxin-mediated developmental programs either independently or collaboratively (Swarup and Péret, 2012). AUX1 plays a key role in root gravitropic response and also functions, together with other LAXs, in lateral root development, root hair development, apical hook development, and phyllotactic patterning (Swarup et al., 2005; Péret et al., 2009; Swarup and Péret, 2012). LAX2 regulates vascular development in cotyledons in Arabidopsis (Péret et al., 2012). LAX3 is involved in lateral root emergence and apical hook development (Swarup et al., 2008; Vandenbussche et al., 2010). All AUX1/LAX members are team players in phyllotactic patterning (Bainbridge et al., 2008; Swarup and Péret, 2012).

The PIN gene family, which has been well characterized in Arabidopsis, includes 8 members (Paponov et al., 2005). PIN proteins contain two predicted highly conserved transmembrane helices at the N- and C-termini, and one highly heterogeneous and hydrophilic loop of various length in the central region (Paponov et al., 2005). A broad range of expression of PIN genes at transcription level has been demonstrated among tissues/cell types and under different growth conditions (Gälweiler et al., 1998; Friml et al., 2002a,b). PIN proteins have been demonstrated to play important roles in embryogenesis (PIN1, PIN4, and PIN7) (Steinmann et al., 1999; Friml et al., 2002a, 2003), organogenesis (PIN1 and PIN4) (Friml et al., 2002a; Benková et al., 2003; Reinhardt et al., 2003), nectary auxin response and short stamen development (PIN6) (Bender et al., 2013), pollen development (PIN5 and PIN8) (Dal Bosco et al., 2012), gravitropism (PIN2 and PIN3) (Müller et al., 1998; Friml et al., 2002b; Ottenschläger et al., 2003), and phototropism (PIN1 and PIN3) (Friml et al., 2002b; Blakeslee et al., 2004).

ABCB proteins belong to the super gene family of ABC transporters and most plant ABCB proteins have been characterized as auxin transporters (Noh et al., 2001; Luschnig, 2002; Geisler et al., 2005; Terasaka et al., 2005). Unlike AUX1/LAXs (auxin influx carriers) and PIN proteins (auxin efflux carriers), ABCB proteins can serve as either auxin transporters with fixed auxin flow directionality (ABCB1, ABCB4, and ABCB19) or facultative auxin importer/exporter (ABCB21) depending on cellular auxin level (Geisler and Murphy, 2006; Cho et al., 2012; Kamimoto et al., 2012). Besides their role in auxin transportation (Cho and Cho, 2013), ABCB proteins exhibited other diverse functions. For example, OsABCB14 functions in both auxin transport and iron homeostasis (Xu et al., 2014), while some other ABCB proteins play a role in secondary metabolite transport, aluminum toxicity response, stomatal response to CO2, and calcium homeostasis (Sasaki et al., 2002; Shitan et al., 2003; Lee et al., 2008).

Recently genome-wide characterization and expression profiling of *AUX/LAX, PIN* and *ABCB* gene families has been reported in many plant species including sorghum, potato, *Populus*, maize, *Medicago truncatula*, and soybean (Shen et al., 2010, 2015; Carraro et al., 2012; Forestan et al., 2012; Roumeliotis et al., 2013; Liu et al., 2014; Wang et al., 2015; Yue et al., 2015; Chai et al., 2016). The observations from these studies that many auxin transporter genes were responsive to hormone and abiotic (or biotic) signals at the transcriptional level suggest that these genes might play a possible role in plants' adaptation to adverse environment by mediating the crosstalk between auxin and other internal signals or external cues. Comprehensive analysis of the PIN gene family in rice showed diversified tissue expression patterns and different responses of the rice PIN genes to hormone stimuli (Wang et al., 2009). The rice *OsPIN3t* was responsive to drought stress, and overexpression of this gene in rice led to improved drought tolerance (Zhang et al., 2012). *OsAUX1* controls many aspects of root development in rice, and responds to Cd and alkaline stresses (Li et al., 2015; Yu et al., 2015; Zhao et al., 2015). However, the underlying mechanisms of how the auxin transporter genes are involved in response to the adverse environmental conditions are still missing.

Rice is a staple food for a large portion of the world population. Although there is an urgent need to provide food security for the growing global population, enhancing rice productivity is very challenging especially under unfavorable environments (Zhang, 2007; Normile, 2008). Taking into account the importance of auxin transporter proteins in plants' growth and development, as well as responses to adverse environment (Swarup and Péret, 2012; Wang et al., 2015; Yue et al., 2015), we conducted a comprehensive study of *OsLAX* and *OsABCB* gene families in rice including phylogenetic analysis, genomic distribution, gene structure, tissue/organ specific expression, expression pattern in response to hormonal stimuli (auxin and ABA) and abiotic stresses (drought and salinity), and *cis*-regulatory element analysis, which lays a foundation for further investigation of their biological functions.

## Materials and methods

### Identification of *OsLAX* auxin influx carriers and *OsABCB* auxin transporters from rice

Putative LAX auxin influx carriers and ABCB auxin transporters in rice were identified by Blast searches against the reference genome at Phytozome v10.3 (http://phytozome.jgi.doe.gov/pz/portal.html) using *Arabidopsis thaliana* AUX1\LAX and ABCB protein sequences as queries, respectively. AUX\LAX and ABCB homologs from maize and sorghum, which were identified by other researchers (Shen et al., 2010; Yue et al., 2015), were used. All identified AUX\LAX and ABCB (Table S1) protein sequences were downloaded for downstream analysis.

### Phylogenetic analysis and chromosomal distribution of *OsLAXs* and *OsABCBs*

Multiple-alignment was performed using Clustal Omega (McWilliam et al., 2013) and the resulting sequence alignments were then used to construct the unrooted phylogenetic tree by the maximum likelihood method with a bootstrap analysis of 1000 replicates using MEGA 5.2 (Tamura et al., 2011). Chromosome localization of each *OsLAX* and *OsABCB* gene in this study was determined based on the position of genes in genome annotation on rice chromosomes. Duplicated genes (with nucleotide sequence identity >90%) were identified using Lasergene software v7.1 (DNASTAR, Madison, USA).

### Gene structure, protein profile, and promoter analysis

The exon-intron structure of each *OsLAX* and *OsABCB* gene was identified using the Gene Structure Display Server (Guo et al., 2007). Protein profiling and stress-related *cis*-regulatory elements analysis were performed as previously described (Chai et al., 2015; Wang et al., 2015).

### Plant growth, treatments and collection of tissues

Rice (*Oryza sativa* L. cv. Pokkali) plants were grown in pots containing a mixture of turface and sand (2:1) in greenhouse under a 16-h light/8-h dark regime with temperature setting at 29°C (light)/26°C (dark). Two-week old rice seedlings were subjected to drought, salinity, auxin (IAA), and ABA treatments. Drought stress was initiated by withholding water and terminated when the relative soil water content reach 69% (for moderate drought stress) and 48% (for severe drought stress) of field capacity. The soil water content of well-watered seedlings (which were used as control plants) was kept at field capacity with the absolute soil water content ranging from 24 to 26%. For salinity stress treatment, rice seedlings were irrigated with 250 mM NaCl and incubated for 1, 5, 24, and 48 h, respectively. For ABA and auxin treatments, two-week old seedlings were sprayed with 200 μM ABA or 10 μM IAA, respectively, and incubated for 0.5, 1, 3, and 5 h, respectively; while a mock treatment without hormone was applied to the control plants. The shoot and root of treated and control seedlings were harvested and frozen in liquid nitrogen and stored at −80°C. All samples (including treated and controls) were collected in biological triplicates. Experiments for drought, salinity, IAA, and ABA were carried out separately. For each of them, three independent experiments were conducted, and in each experiment a collection of samples from four rice seedlings for each treatment (or control) was used as one biological replicate.

### RNA isolation, quantitative RT-PCR (qRT-PCR), microarray data collection, and heat map construction

RNA was isolated using Trizol® Reagent (Thermo Fisher Scientific, Waltham, MA, USA) and then treated with DNAse from TURBO DNA-free™ Kit (Thermo Fisher Scientific, Waltham, MA, USA) to remove possible DNA contamination following the instructions of the manufacturer. Primers for qRT-PCR were designed using Primer3web program (http://bioinfo.ut.ee/primer3). Rice *OsActin1* (LOC_Os03g50885) was used as internal reference gene for normalization of gene expression. cDNA synthesis, qRT-PCR reaction setup, data analysis, and heat map construction were conducted following the method as previously described (Chai et al., 2015). Primers for qRT-PCR were listed in Table S2. Publicly available rice microarray data were retrieved for tissue specific expression of *OsLAXs* and *OsABCBs* (http://bar.utoronto.ca/welcome.htm) (Jain et al., 2007; Patel et al., 2012).

## Results and discussion

### Genome-wide identification and phylogenetic analysis of *OsLAXs* and *OsABCBs*

A total of 5 putative *OsLAX*s and 22 putative *OsABCB*s were identified in the rice genome by using Arabidopsis AUX1/LAX, and AtABCB protein sequences as queries to BLAST search against rice genome (v7.0), respectively (Table S1). The number of *OsLAX* genes in rice (5) is similar to that of maize (5), sorghum (5) and Arabidopsis (4), but much less than that of soybean (15) (Chai et al., 2016). The number of *ABCB* gene of rice (22) is comparable with that of Arabidopsis (21) and sorghum (24), but less than that of maize (35).

**Figure 1:**
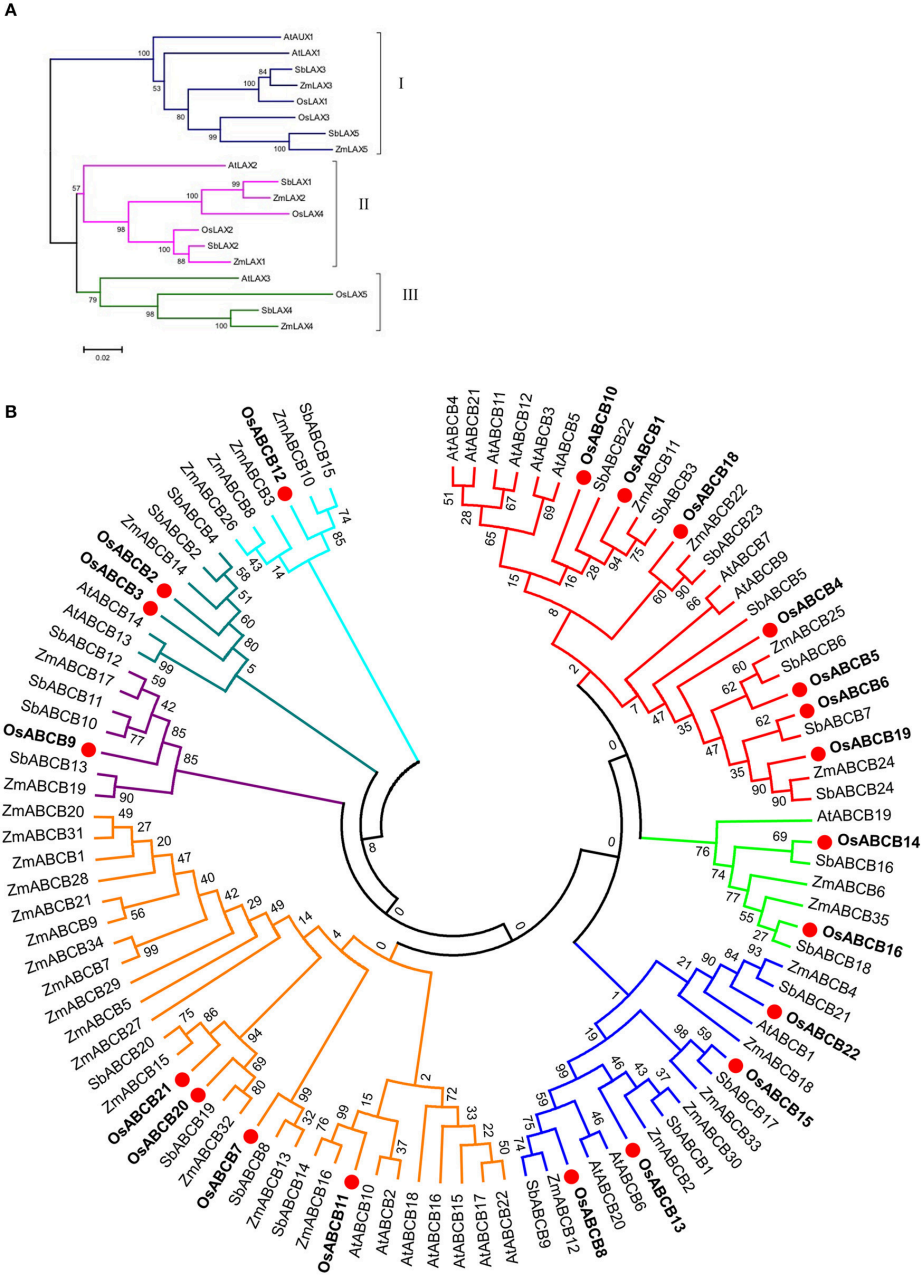
**Phylogenetic analysis of LAXs (A) and ABCBs (B) auxin transporter protein families from rice, Arabidopsis, sorghum, and maize**. The protein sequences of sorghum and maize were retrieved from Phytozome (v10.3) based on recent publications (Shen et al., 2010; Yue et al., 2015). The phylogenetic trees were constructed using Mega5.2 program (Tamura et al., 2011).

To predict the evolutionary relationships and functions of OsLAXs and OsABCBs, unrooted phylogenetic trees were constructed using full-length protein sequences of these two types of auxin transporter gene families of rice, Arabidopsis, maize, and sorghum (Figure 1). The LAX proteins were classified into three subfamilies with 8 members in group I, 7 in group II, and 4 in group III, respectively (Figure 1A). The OsLAXs showed closer evolutionary relationship to the other monocots (maize and sorghum) compared with those from the dicot plant Arabidopsis. OsLAX1, also named as OsAUX1 in other publications, shares high homology with AtAUX1, plays an important role in rice root development, including lateral root initiation and the primary root and root hair elongation, and is responsive to Cd and alkaline stresses and nitrogen nutrition status (Song et al., 2013; Li et al., 2015; Yu et al., 2015; Zhao et al., 2015).

The phylogenetic tree divided the 100 ABCB proteins from the 4 species into 7 groups, with rice member(s) in every group (Figure 1B). Five out of 7 groups contained both dicot and monocot members and the other two groups were monocot-specific, which had only members from grass family (the groups with OsABCB9 and OsABCB12). Four Arabidopsis ABCB proteins (AtABCB1, AtABCB4, AtABCB19, and AtABCB21) have been characterized as auxin transporters (Geisler and Murphy, 2006; Cho et al., 2012; Kamimoto et al., 2012). In rice, only OsABCB14 (AtABCB1 and AtABCB19 homologs) was reported to function in auxin transport and iron homeostasis (Xu et al., 2014). The vast majority of ABCB proteins of rice and other species remain to be investigated.

### Chromosomal distribution, gene structure, and protein profiles of *OsLAXs* and *OsABCBs*

**Figure 2:**
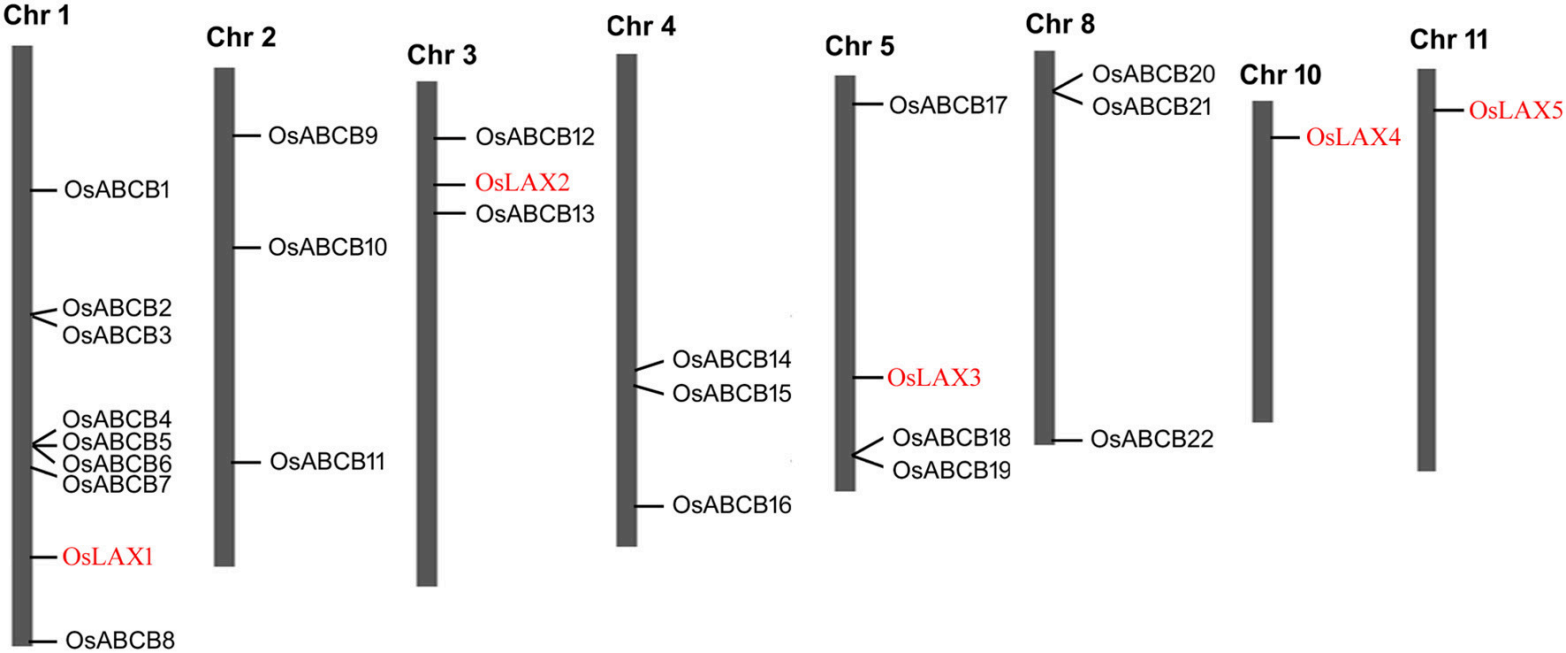
**Chromosomal distribution of *OsLAXs* and *OsABCBs***. The position of each gene was determined based on the start position of each gene.

The five *OsLAX*s and 22 *OsABCB*s were mapped onto 8 rice chromosomes based on the start position of each gene (Figure 2). The *OsLAX* genes were distributed on chromosomes 1, 3, 5, 10, and 11 with one gene on each chromosome. The *OsABCB* genes were not evenly mapped onto 6 chromosomes, with 2 genes on chromosome 3, 3 genes each on chromosomes 2, 4, 5, and 8, and 7 genes on chromosome 1.

**Figure 3:**
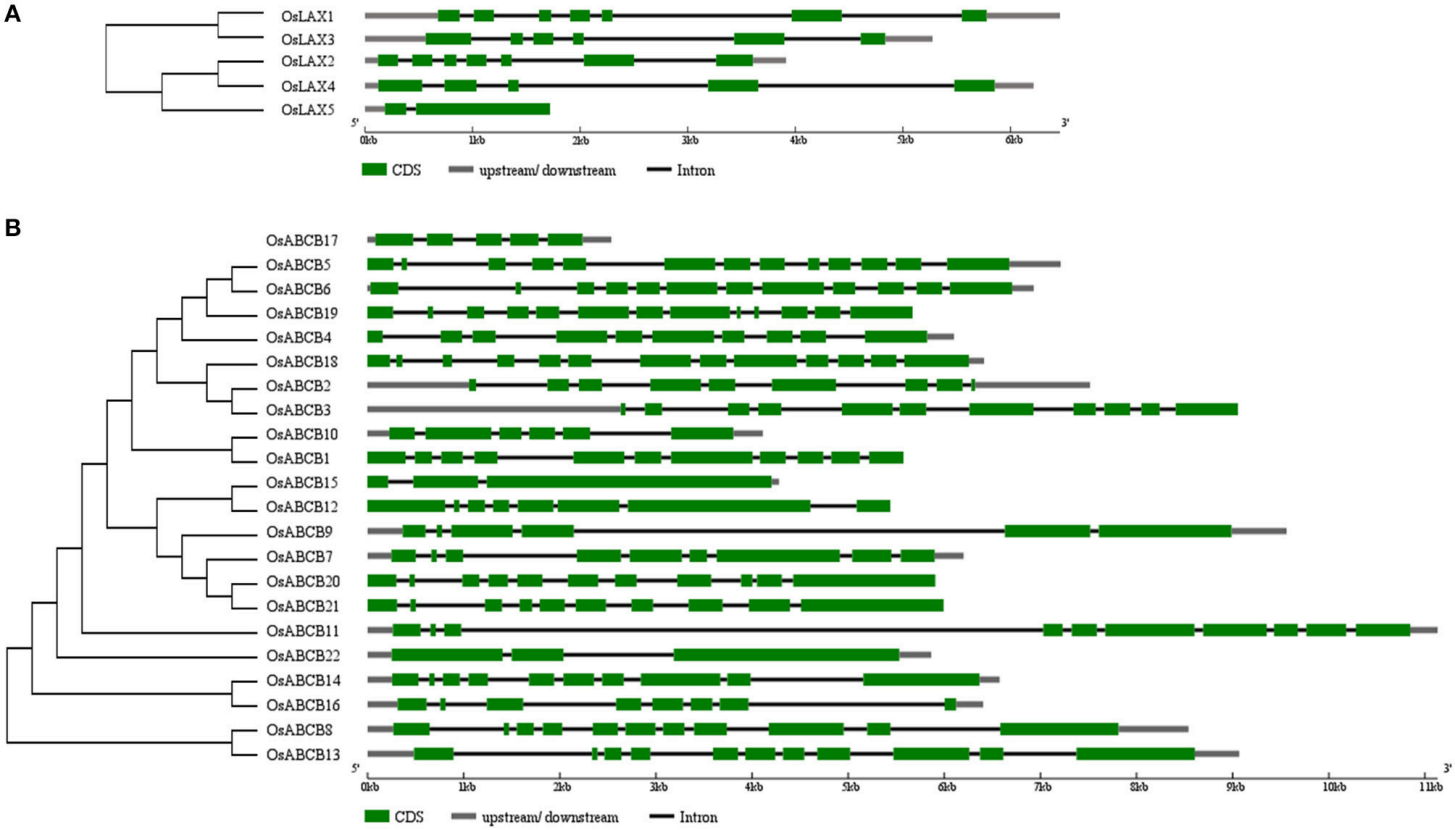
**Phylogenetic analysis and gene structures of ***OsLAXs*** (A) and ***OsABCBs*** (B)**.

Rice has experienced ancient whole-genome duplication, recent segment duplication, and is now experiencing large-scale individual gene duplication (Yu et al., 2005). However, no authentic duplicate gene was identified in either *OsLAX*s or *OsABCB*s due to their nucleotide/amino acid identities (Tables S3, S4) lower than the threshold (90%) required to be defined as duplicate genes. Genes that showed closest evolutionary relationship (Figure 3) were here considered as “gene pair.” Two “gene pairs” were identified in *OsLAX* family (*OsLAX1-OsLAX3* and *OsLAX2*-*OsLAX4*) and 7 gene pairs were identified in *OsABCB* family (*OsABCB5-OsABCB6, OsABCB2-OsABCB3, OsABCB1-OsABCB10, OsABCB12-OsABCB15, OsABCB20-OsABCB21, OsABCB14-OsABCB16*, and *OsABCB8-OsABCB13*).

The gene structure of *AUX/LAX*s is relatively conserved within grass family. The exon number of *OsLAX*s varied between 2 and 7 (Figure 3), while those of maize and sorghum range from 3 to 8 (Shen et al., 2010; Yue et al., 2015). Compared with auxin influx transporter in monocots, the exon-intron organization of dicots counterparts was much more conserved with 6–8 exons in Arabidopsis and 8 exons in soybean (Chai et al., 2016). The gene structure of *OsABCB*s varies greatly with each gene containing 2–13 exons. The variation in gene structure of *ABCB* genes was also identified in maize and sorghum (Shen et al., 2010; Yue et al., 2015). For both *OsLAX*s and *OsABCB*s, the total intron size is the primary factor determining the size difference of genomic gene sequence.

The deduced OsLAX proteins were 480~547 amino acids long, with molecular weight of 52~60 kDa and isoelectric point (PI) of 8.5~9.2 (Table S5). Knowing the subcellular localization of proteins is very helpful for understanding their functions. The subcellular localizations of OsLAX proteins were predicted by WoLF PSORT (Horton et al., 2007). OsLAX1-3 was predicted to be localized in plasma membrane, while OsLAX4 and 5 had a possible localization in cytoplasm. Consistent with the subcellular localization prediction, topology analysis using TMHMM Server v. 2.0 (Krogh et al., 2001) indicated that OsLAXs contained 9–11 transmembrane helices constituting the core permease region (Table S5, Figure S1A). Auxin influx transporter of other plants (Arabidopsis, soybean, maize, and sorghum) showed more conserved domain topology with 10 transmembrane spanning domains (Chai et al., 2016). However, the 11 transmembrane spanning domains of the Arabidopsis AUX1 were determined by using an approach combining prediction program and experimental method (Swarup et al., 2004). The topology of OsLAXs could be further confirmed using the same approach.

The OsABCBs showed a broad range in terms of protein properties (Table S5). They range from 524 (OsABCB17) to 1482 amino acids (OsABCB12) in length, 56 (OsABCB17) to 158 kDa (OsABCB12) in molecular weight, and 5.7 (OsABCB21) to 9.3 (OsABCB11) for PI. The majority of OsABCBs were predicted plasma membrane-localized, except for OsABCB8 and OsABCB22 with localization in cytoplasm, and OsABCB12 and OsABCB17 with localization in chloroplast. The OsABCBs comprised 4–13 transmembrane spanning domains and could be divided into two groups based on topologic characteristics: most members showed two clusters of transmembrane helices at N- and C- termini linked by a central loop in various lengths and only three members (OsABCB10, OsABCB16, and OsABCB17) contained only one cluster of transmembrane helices (Table S5, Figure S1B). For most OsABCBs, the transmembrane helices at N- and C- termini were highly conserved and the loops between them were of high heterogeneity (Figure S1B). The ABCB proteins can serve as auxin influx/efflux transporter and transporters for secondary metabolite as well as iron (Terasaka et al., 2005; Cho et al., 2007; Lee et al., 2008; Xu et al., 2014). However, there is no sufficient evidence to establish a clear relationship between topologic structure of ABCB proteins and their specific functions.

### Tissue-specific expression of *OsLAXs* and *OsABCBs*

**Figure 4:**
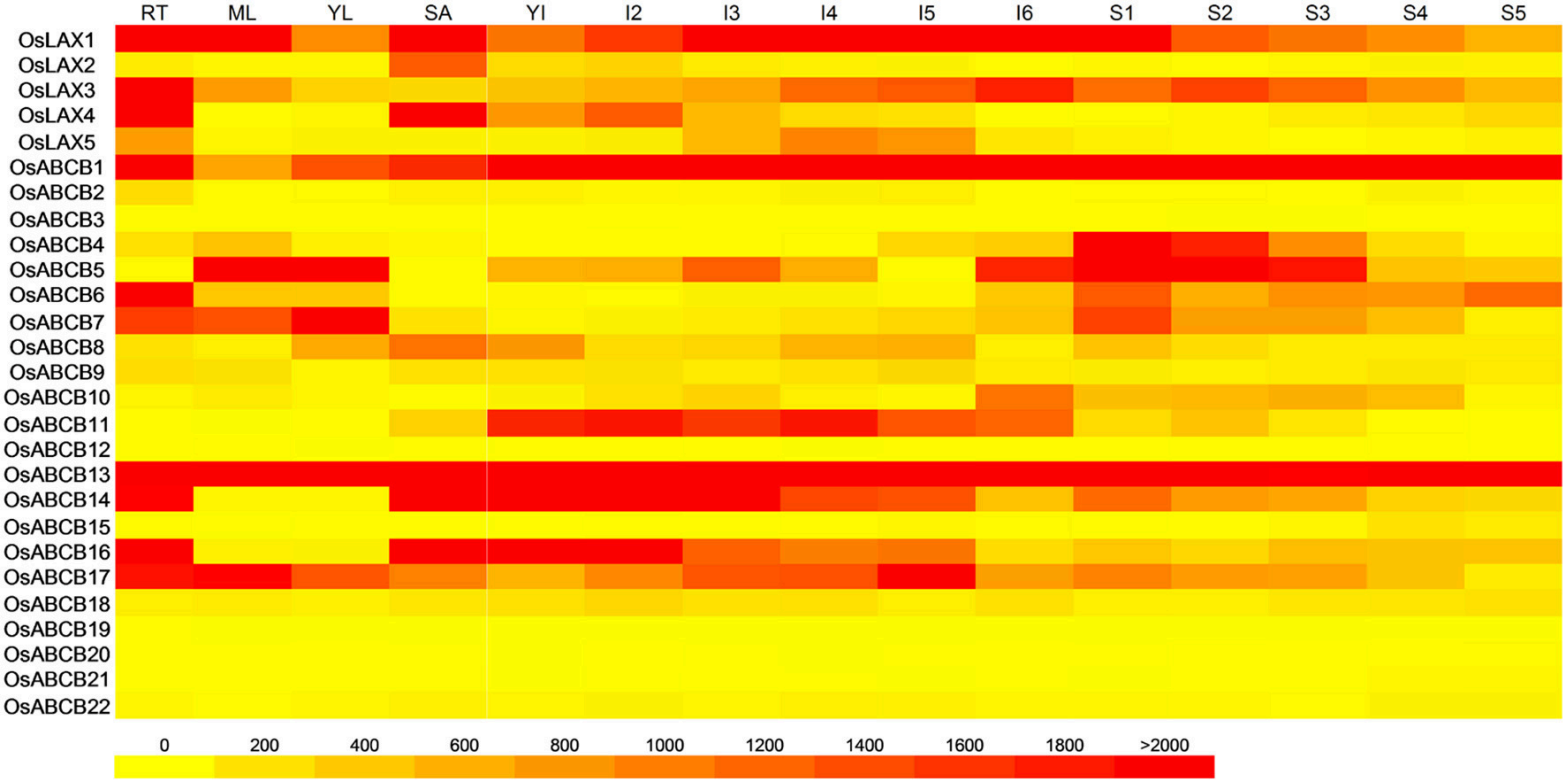
**Tissue/organ specific gene expression patterns of *OsLAXs* and *OsABCBs***. The heat map was constructed using publicly available microarray data of rice (Jain et al., 2007; Patel et al., 2012). RT, roots; ML, mature leaves; YL, young leaves; SA, shoot apical meristem; YI, young inflorescence; I2–I6, inflorescence from stage 2 to stage 6; S1–S5, developing seed from stage1 to stage5.

Tissue-specific expression patterns of genes could contribute significantly to better understanding of their biological roles. A heat map showing the gene expressions of *OsLAX*s and *OsABCB*s in 6 tissues/organs including root, mature leaf, young leaf, shoot apex meristem (SAM), developing panicle (P1–P5), and seed (S1–S5) at different stages was constructed using publicly available rice microarray data (Jain et al., 2007; Patel et al., 2012). The *OsLAX*s showed both ubiquitous and tissue-specific expression patterns (Figure 4). *OsLAX1* and *OsLAX3* were expressed in all tissues examined; *OsLAX2* was exclusively expressed in SAM; *OsLAX4* was predominantly expressed in roots and SAM, and *OsLAX5* was preferably expressed in young panicles. The distinct expression patterns of *OsLAX*s suggested their functions in these tissues/organs.

For *OsABCB*s, more diverse expression patterns were found (Figure 4). Highly expressed *OsABCB1* and *OsABCB13*, along with *OsABCB17*, were expressed in all tissues examined. *OsABCB14* and *OsABCB16* were expressed in all tissues except for leaves. *OsABCB4* was most abundantly expressed in developing seeds while *OsABCB10* and *OsABCB11* were dominantly expressed in panicles. Genes not ubiquitously expressed but highly expressed in more than one tissue were *OsABCB5* (leaves, panicle, and seed)*, OsABCB6* (root and seed)*, OsABCB7* (root, leaf, and seed) and *OsABCB8* (young leaf, panicle, SAM, and seed). Several genes showed low or no expression in any tissue.

It is worth to note that some “gene pairs” identified in this study (Figure 3) exhibited diverse and interesting expression patterns (Figure 4). For example, *OsABCB14* and *OsABCB16* showed very similar expression pattern, which suggested functional redundancy; *OsABCB5* and *OsABCB6* demonstrated a complementary expression mode, indicating possible functional complementation; for the two gene pairs *OsABCB1-OsABCB10* and *OsABCB13-OsABCB8*, expression of one member *(OsABCB1* and *OsABCB13*) was high in almost every tissue, while the expression of other member (*OsABCB10* and *OsABCB8*) was generally low or absent, which suggested possible gain or loss of function as a result of evolution.

### Expression profiles of *OsLAXs* and *OsABCBs* under drought and salt stresses

**Figure 5:**
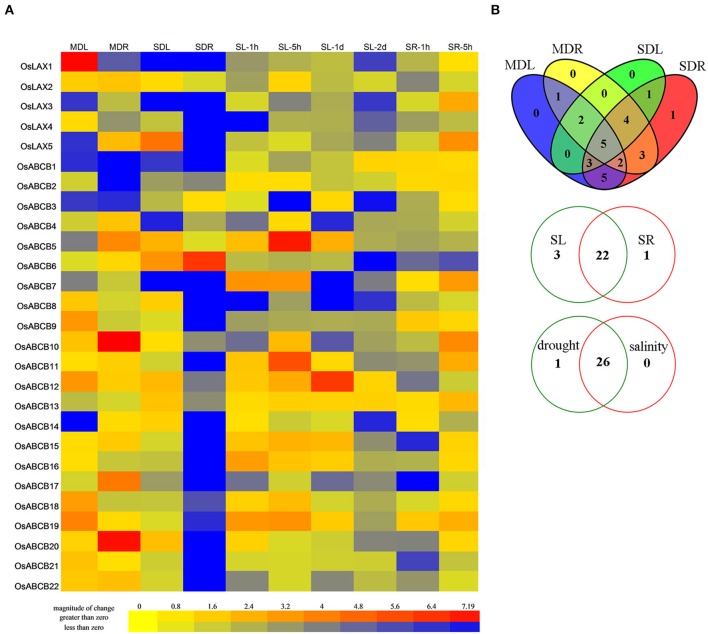
**Expression profiles of ***OsLAXs*** and ***OsABCBs*** under drought and salinity stresses**. **(A)** Fold change of *OsLAXs* and *OsABCBs* gene expression in shoots and roots under drought and salinity stresses. Data presented here are means of fold changes of qRT-PCR under moderate and severe drought, and salinity stress with biological triplicates and two technical replicates. MDL, moderate drought stressed leaves; MDR, moderate drought stressed roots; SDL, severe drought stressed leaves; SDR, severe drought stressed roots; SL-1h, leaves at 1 h after imposition of salinity stress; SL-5h, leaves at 5 h after imposition of salinity stress; SL-1d, leaves at 1day after imposition of salinity stress; SL-2d, leaves at 2 days after imposition of salinity stress; SR-1h, roots at 1 h after imposition of salinity stress; SR-5h, roots at 5 h after imposition of salinity stress; SR-1d, roots at 1 day after imposition of salinity stress; SR-2d, roots at 2 days after imposition of salinity stress. **(B)** Venn diagram analysis of data in **(A)**. Summary of *OsLAXs* and *OsABCBs* gene expression in shoots and roots under drought and salinity stresses.

Drought and salinity are two major abiotic stresses that significantly reduce rice production worldwide (Dolferus et al., 2011; Roy et al., 2011). Auxin transporters from several species were shown transcriptionally responsive to abiotic stresses, which might lead to plants' adaptation to these adverse conditions (Habets and Offringa, 2014; Wang et al., 2015). In order to discover the possible roles of 5 *OsLAXs* and 22 *OsABCBs* in abiotic stress response/adaptation, their expression profiles at transcription level were investigated by qRT-PCR (Figure 5). Genes with absolute fold change ≥2 and *p*-value < 0.05 by student's *t*-test were considered as responsive genes. The expressions of all the 5 *OsLAXs* and 21 *OsABCBs* were responsive to both drought and salinity, except for *OsABCB2*, which was only responsive to drought stress. Under drought and/or salt stress conditions, most *OsLAXs* and *OsABCBs* were down regulated while 3 *OsLAXs* (*OsLAX1, 3*, and *5*) and 14 *OsABCBs* (*OsABCB5-7, 9-13*, and *15-20)* were up regulated in certain tissue and/or under certain treatments. *OsLAX5* and *OsABCB5, 12, 18*, and *19* were up regulated under both drought and salt stress. Under moderate drought stress, the number of differentially expressed genes in roots was similar to that in leaves (17 vs. 18), but much greater under severe drought stress (24 vs. 15). Interestingly, more genes were regulated in roots than in leaves (15 vs. 10) by both drought treatments. Upon salt stress, 22 out of 27 genes (*OsLAXs* and *OsABCBs*) were differentially expressed in both leaves and roots, and three genes were differentially expressed in leaves, and one gene was differentially expressed in roots.

Since most *OsLAXs* and *OsABCBs* were transcriptionally regulated by drought and/or salinity stresses, these genes may play an important role in abiotic stress response and adaptation. The diverse responsive expression patterns of *OsLAX* and *OsABCB* genes also indicated the complexity of molecular regulatory network underlying these adaptive processes. The role of auxin transporters (especially AUX1/LAX and PINs) in auxin transportation and related biological processes has been well demonstrated in model plant Arabidopsis (Paponov et al., 2005; Swarup and Péret, 2012). Although the close relationship between auxin transporter genes and abiotic stresses has been reported in many plants (Shen et al., 2010; Wang et al., 2015; Yue et al., 2015), the exact functions of the auxin transporter genes in abiotic stress adaptation remain largely unexplored. Auxin/auxin transport has been shown to play a role in oxidative stress tolerance caused by arsenite (Krishnamurthy and Rathinasabapathi, 2013). In addition, the Arabidopsis auxin influx carrier mutant *aux1* was more sensitive to drought, salinity, and high temperature stresses. *OsAUX1* (*OsLAX1*), the rice ortholog of *AUX1*, was responsive to Cd and alkaline stresses (Li et al., 2015; Yu et al., 2015). Current evidence suggests that auxin transporters may be directly or indirectly involved in abiotic stress adaptation/tolerance (Zhang et al., 2012), likely through regulation of auxin transport/redistribution.

### Expression profiles of OsLAXs and OsABCBs under auxin and ABA treatment

**Figure 6:**
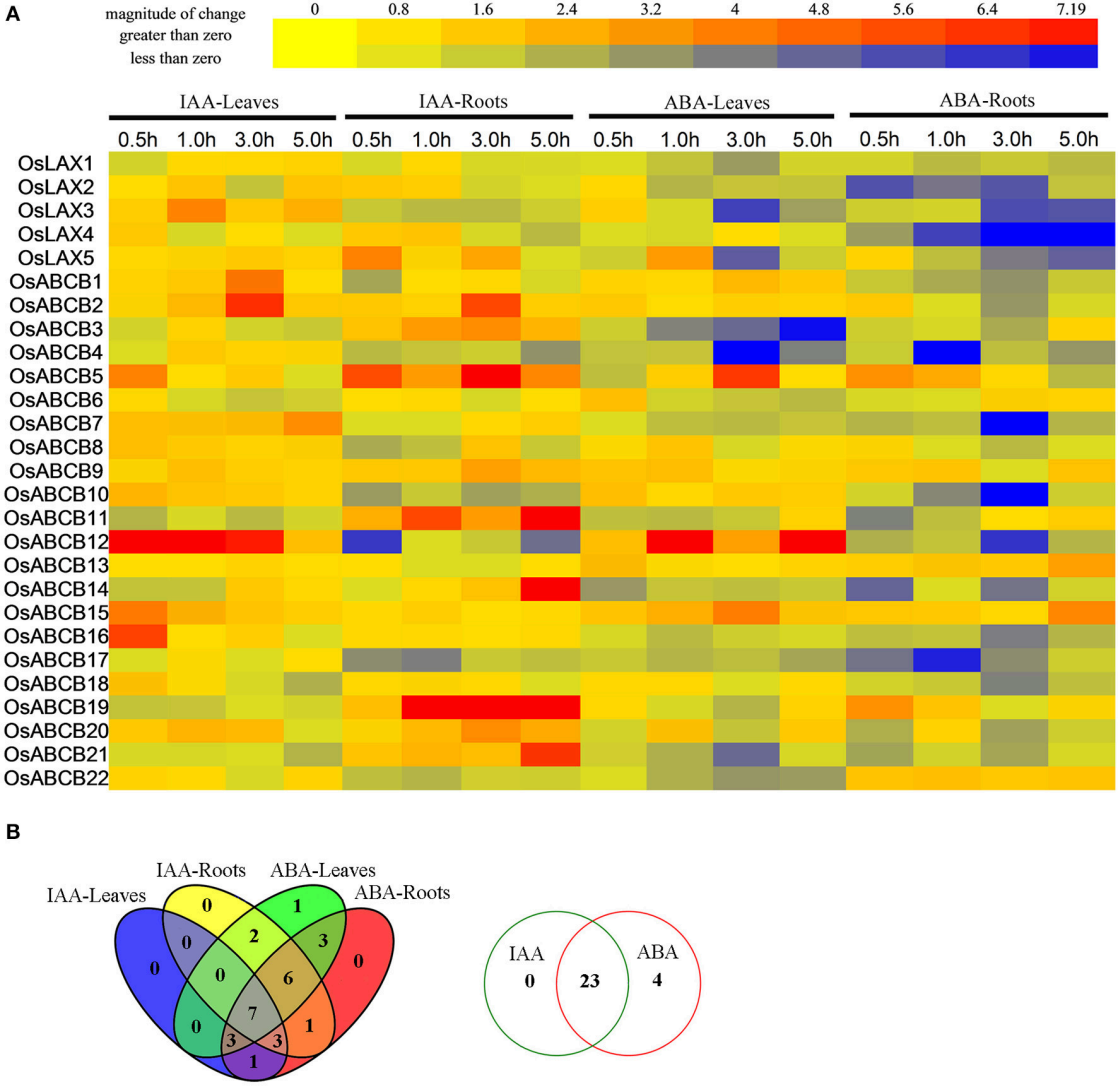
**Expression profiles of ***OsLAXs*** and ***OsABCBs*** under IAA and ABA stimuli**. **(A)** Fold change of *OsLAXs* and *OsABCBs* gene expression in shoots and roots under auxin and ABA treatments. Data presented here are means of fold changes of qRT-PCR under auxin (10 μM IAA) and ABA (200 μM) treatments with biological triplicates and two technical replicates. IAA-L, IAA treated leaves; IAA-R; IAA treated roots; ABA-L, ABA treated leaves; ABA-R, ABA treated roots. **(B)** Venn diagram analysis of data in **(A)**. Summary of *OsLAXs* and *OsABCBs* gene expression in shoots and roots under auxin and ABA treatments.

Auxin is a multifunctional plant hormone, which plays a fundamental role in coordination of various developmental processes, and an emerging role in mediating environmental adaptation in plants (Kazan, 2013). ABA, as the plant “stress hormone,” is essential for plants in responding to abiotic stresses (drought, salinity, and cold, etc.), in addition to their multiple roles in plants' development (Mehrotra et al., 2014). qRT-PCR was performed to explore how these two plant hormones affect gene expression of *OsLAX*s and *OsABCB*s in leaves and roots of rice seedlings (Figure 6). Overall, most genes were responsive to one or both hormones. Twenty-three genes (3 *OsLAX*s and 20 *OsABCB*s) were differentially expressed upon auxin treatment, with 4 exclusively in leaves, 9 specifically in roots, and 10 in both leaves and roots. Among these auxin-regulated genes, most were up regulated and only 4 genes were down regulated at some time points of treatment. Since most *OsLAXs* and *OsABCBs* were regulated by auxin and were sub-localized in plasma membrane (Table S5), they are probably involved in auxin transport in rice.

Surprisingly, all *OsLAX*s and *OsABCB*s were responsive to ABA cue, with 19 genes differentially expressed in both leaves and roots, 3 in leaves, and 5 in roots. Most genes were repressed by ABA in either leaves or roots. Interestingly, 3 *OsLAX*s and 20 *OsABCB*s were regulated by both auxin and ABA, suggesting their involvement in both auxin and ABA signaling pathways.

Besides the diverse and critical roles during plant development under optimal growth environments (Durbak et al., 2012), hormone interactions/crosstalks and hormone balance are crucial in plant adaptation to stressful environmental conditions (Peleg and Blumwald, 2011). Under water deficit conditions, many hormone-related genes were differentially regulated, as revealed in transcriptomic and/or metabolomic studies conducted in rice, wheat, and soybean (Krugman et al., 2011; Wang et al., 2011; Le et al., 2012; Song et al., 2016). Recent studies indicate that auxin is the central regulator in cold stress response or a mediator or organizer of environmental adaptation in plants (Kazan, 2013; Lee and Cho, 2013; Rahman, 2013). Therefore, the auxin system works as a central integrator of intrinsic and extrinsic signals (Kieffer et al., 2010). Regulation of auxin uneven distribution within tissues/organs and throughout the whole plant body via auxin transporters is one important strategy to execute the auxin function in controlling various plant developmental processes (Michniewicz et al., 2007; Zažimalová et al., 2010), as well as in response to stressful environments. It has been found that auxin concentration or distribution was regulated by water stress or ABA treatment in rice, Arabidopsis, and soybean, and some PIN genes might be involved in those processes (Xu et al., 2013; Wang et al., 2015). Our study indicates many *OsLAX*s and *OsABCB*s might have such a role in rice. Notably, more *OsLAX* and *OsABCB* genes were responsive to severe drought stress and IAA treatments in roots than in leaves, and some genes were oppositely regulated in the two tissues by the same treatments, suggesting their role in different functions in different tissues under these conditions (Figures 5, 6). Synergistic action of these *OsLAXs* and *OsABCBs*, together with *OsPIN*s, might contribute to the dynamic auxin redistribution within the same tissues and/or between shoots and roots, and thereby plant growth adjustment under these conditions. In Arabidopsis and rice, alteration of auxin accumulation or distribution through overexpression of an IAA biosynthetic gene (*YUCCA7*) or auxin transporter gene (*OsPIN3t*) led to drought tolerant phenotype (Lee et al., 2012; Zhang et al., 2012). Further functional characterization in heterologous expression system and overexpression/knock-out transgenic studies in plants will help to elucidate the specific gene functions.

Many auxin transporter genes have been shown to be responsive to a number of plant hormones (Shen et al., 2010; Wang et al., 2015; Yue et al., 2015). Crosstalks among different plant hormones occur in many aspects of plant growth and development, facilitating resource redistribution for these processes by coordinating activities of all players. The interplay between auxin and ABA signaling pathway was well exemplified by the synergistic promotion of root growth in rice and Arabidopsis under water stress (Xu et al., 2013). Our results revealed that most *OsLAXs* and *OsABCB*s were regulated by these two plant hormones suggesting their role in auxin and ABA related biological processes. Since the major function of auxin transporters is auxin transport, which was reflected from literature so far, the involvement of *OsLAXs* and *OsABCB*s in developmental and adaptive programs was probably through regulating auxin distribution. However, it will be interesting to explore new functions of *OsLAXs* and *OsABCB*s in various processes.

### Analysis of *cis*-regulatory elements in promoters of OsLAXs and OsABCBs

**Figure 7:**
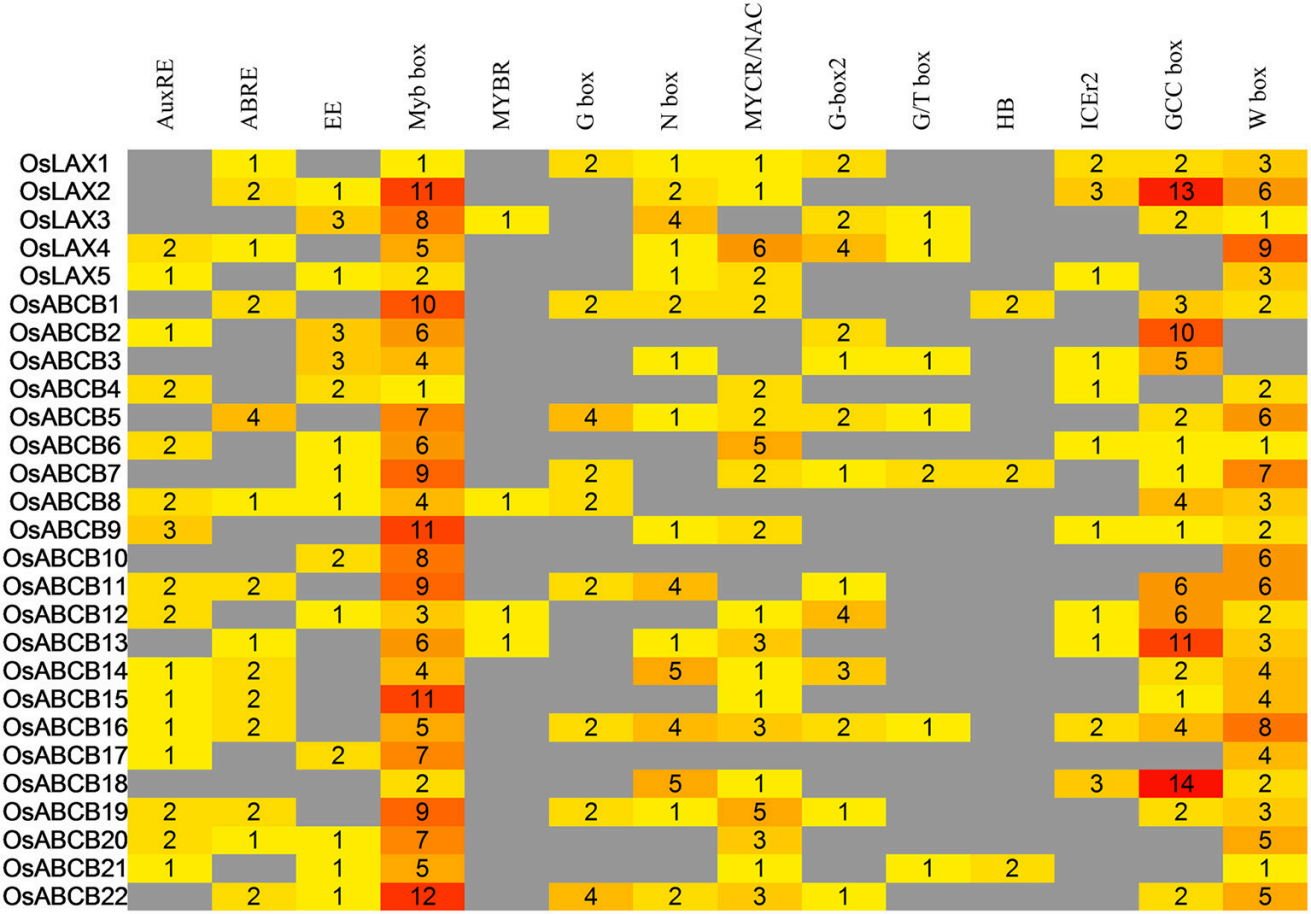
**Analysis of auxin responsive and stress-related ***cis***-regulatory elements in the 2-kb promoter regions of ***OsLAXs*** and ***OsABCBs*****.

*Cis*-regulatory elements (CREs) in non-coding regions such as promoters are determining factors of transcriptional regulation in multicellular organisms (Siepel and Arbiza, 2014; Henry et al., 2015). The diversity of expression patterns *OsLAX*s and *OsABCB*s in different tissues/organs, and in response to abiotic stresses (drought and salinity) as well as hormonal stimuli (auxin and ABA) prompted us to check if any responsive CREs reside in promoter regions. Promoters (~2000 bp) of *OsLAX*s and *OsABCB*s retrieved from phytozome 10.3 were subject to CRE analysis. A total of 604 putative CREs were identified including auxin response elements (AuxREs), ABA responsive elements (ABREs) and other abiotic and biotic responsive elements (Figure 7). The auxin responsive and abiotic stress responsive elements could account for the strong response of *OsLAX*s and *OsABCB*s to the plant hormones and abiotic stresses. Some *OsLAX*s and *OsABCB*s contain cold stress (ICEr2) and biotic stresses (W box) responsive CREs, indicating their involvement in adaptation to these stresses. The CREs are binding sites of transcription factors including ARFs, Mybs, bHLHs, bZIPs, WRKYs, AP2/ERFs, NACs, and homeodomain proteins. Therefore, *OsLAXs* and *OsABCB*s might be regulated by many transcription factors leading to very complex regulatory networks.

In summary, two rice auxin transporter gene families, *OsLAXs* and *OsABCB*s, were comprehensively analyzed, including chromosomal distribution, gene structure, protein topology, phylogenetic relationship, expression profiling under abiotic stresses and phytohormone stimuli, as well as CRE prediction. Responsiveness of *OsLAXs* and *OsABCB*s to auxin stimulus and transmembrane features of their corresponding proteins suggested that they might be true auxin transporters. Furthermore, most *OsLAXs* and *OsABCB*s genes responded to drought, salinity, and ABA treatment, indicating their important role as the mediator of crosstalks among abiotic stresses and hormonal signaling pathways. Promoter CRE analysis revealed possible regulation by a number of transcription factors and involvement in biotic stresses. Our results provide valuable information for further investigation of *OsLAXs* and *OsABCBs* to facilitate development of stress tolerant rice varieties.

## Author contributions

CC and PS conceived and designed the experiment. CC conducted the experiment, analyzed the data, and wrote the manuscript. CC and PS critically revised the manuscript. CC and PS read and approved the manuscript.

### Conflict of interest statement

The authors declare that the research was conducted in the absence of any commercial or financial relationships that could be construed as a potential conflict of interest.

## Supplementary material

Figure S1 **Transmembrane topology analysis of OsLAXs (A) and OsABCBs (B) proteins**. The transmembrane protein topology was predicted by using the TMHHM Server v2.0 (Krogh et al., 2001). The predicted transmembrane helices were shown as red peaks on the top.

Table S1 **Protein sequences used in the phylogenetic analysis**.

Table S2 **Primers used in qPCR analyses**.

Table S3 **Percent ORF nucleotide (bottom-left) and amino acid (up-right, bold) identities among OsLAXs**.

Table S4 **Percent ORF nucleotide (bottom-left) and amino acid (up-right, bold) identities among OsABCBs**.

Table S5 **Gene information of *OsLAXs* and *OsABCBs***.
