# Supplementary figures and images for: Comprehensive Analysis and Expression Profiling of the OsLAX and OsABCB Auxin Transporter Gene Families in Rice (Oryza sativa) under Phytohormone Stimuli and Abiotic Stresses
Source: Front Plant Sci. 2016 May 3;7:593. doi: 10.3389/fpls.2016.00593 (PMC4853607; doi:10.3389/fpls.2016.00593)

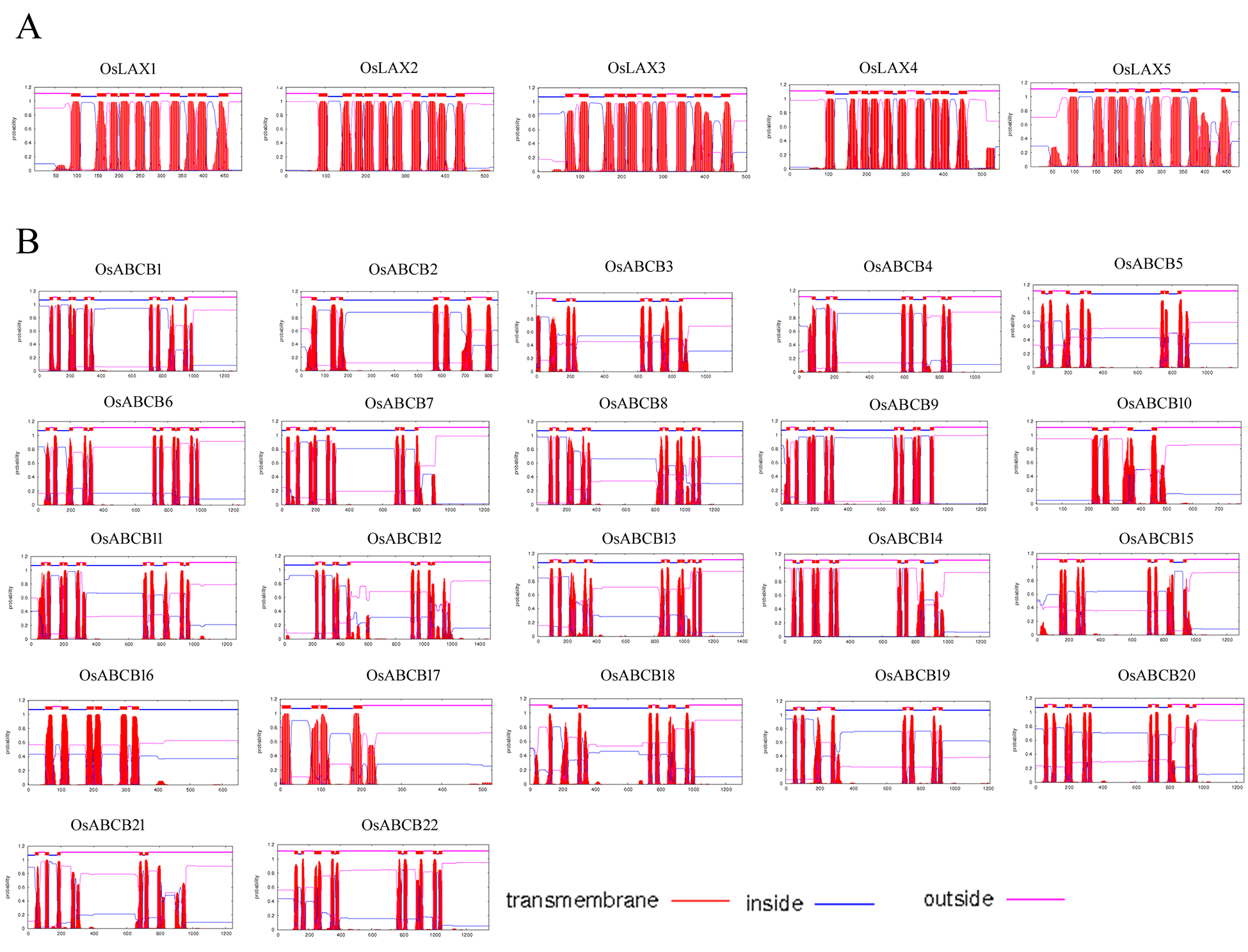

Supplement: Figure S1 — Transmembrane topology analysis of OsLAXs (A) and OsABCBs (B) proteins. The transmembrane protein topology was predicted by using the TMHHM Server v2.0 (Krogh et al., 2001). The predicted transmembrane helices were shown as red peaks on the top. [file Image1.TIF]
