# Supplementary material for: Comprehensive Analysis and Expression Profiling of the OsLAX and OsABCB Auxin Transporter Gene Families in Rice (Oryza sativa) under Phytohormone Stimuli and Abiotic Stresses
Source: Front Plant Sci. 2016 May 3;7:593. doi: 10.3389/fpls.2016.00593 (PMC4853607; doi:10.3389/fpls.2016.00593)
Supplement: Table S1 — Protein sequences used in the phylogenetic analysis. [file Table1.DOCX]

| **Table S1.** Protein sequences used in the phylogenetic relationship analysis**.** | | | | |  |
| --- | --- | --- | --- | --- | --- |
|  |  |  |  |  |  |
| **Species** | **Gene name** | **Locus ID** | **Synonyms name** |  |  |
| Arabidopsis | AtAUX1 | At2g38120 |  |  |  |
|  | AtLAX1 | At5g01240 |  |  |  |
|  | AtLAX2 | At2g21050 |  |  |  |
|  | AtLAX3 | At1g77690 |  |  |  |
|  | AtABCB1 | At2g36910 | AtPGP1 |  |  |
|  | AtABCB2 | At4g25960 | AtPGP2 |  |  |
|  | AtABCB3 | At4g01820 | AtPGP3 |  |  |
|  | AtABCB4 | At2g47000 | AtPGP4 |  |  |
|  | AtABCB5 | At4g01830 | AtPGP5 |  |  |
|  | AtABCB6 | At2g39480 | AtPGP6 |  |  |
|  | AtABCB7 | At5g46540 | AtPGP7 |  |  |
|  | AtABCB9 | At4g18050 | AtPGP9 |  |  |
|  | AtABCB10 | At1g10680 | AtPGP10 |  |  |
|  | AtABCB11 | At1g02520 | AtPGP11 |  |  |
|  | AtABCB12 | At1g02530 | AtPGP12 |  |  |
|  | AtABCB13 | At1g27940 | AtPGP13 |  |  |
|  | AtABCB14 | At1g28010 | AtPGP14 |  |  |
|  | AtABCB15 | At3g28345 | AtPGP15 |  |  |
|  | AtABCB16 | At3g28360 | AtPGP16 |  |  |
|  | AtABCB17 | At3g28380 | AtPGP17 |  |  |
|  | AtABCB18 | At3g28390 | AtPGP18 |  |  |
|  | AtABCB19 | At3g28860 | AtPGP19 |  |  |
|  | AtABCB20 | At3g55320 | AtPGP20 |  |  |
|  | AtABCB21 | At3g62150 | AtPGP21 |  |  |
|  | AtABCB22 | At3g28415 | AtPGP22 |  |  |
| Rice | OsLAX1 | LOC_Os01g63770 |  |  |  |
|  | OsLAX2 | LOC_Os03g14080 |  |  |  |
|  | OsLAX3 | LOC_Os05g37470 |  |  |  |
|  | OsLAX4 | LOC_Os10g05690 |  |  |  |
|  | OsLAX5 | LOC_Os11g06820 |  |  |  |
|  | OsABCB1 | LOC_Os01g18670 | OsPGP1 |  |  |
|  | OsABCB2 | LOC_Os01g34970 | OsPGP2 |  |  |
|  | OsABCB3 | LOC_Os01g35030 | OsPGP3 |  |  |
|  | OsABCB4 | LOC_Os01g50080 | OsPGP4 |  |  |
|  | OsABCB5 | LOC_Os01g50100 | OsPGP5 |  |  |
|  | OsABCB6 | LOC_Os01g50160 | OsPGP6 |  |  |
|  | OsABCB7 | LOC_Os01g52550 | OsPGP7 |  |  |
|  | OsABCB8 | LOC_Os01g74470 | OsPGP8 |  |  |
|  | OsABCB9 | LOC_Os02g09720 | OsPGP9 |  |  |
|  | OsABCB10 | LOC_Os02g21750 | OsPGP10 |  |  |
|  | OsABCB11 | LOC_Os02g46680 | OsPGP11 |  |  |
|  | OsABCB12 | LOC_Os03g08380 | OsPGP12 |  |  |
|  | OsABCB13 | LOC_Os03g17180 | OsPGP13 |  |  |
|  | OsABCB14 | LOC_Os04g38570 | OsPGP14 |  |  |
|  | OsABCB15 | LOC_Os04g40570 | OsPGP15 |  |  |
|  | OsABCB16 | LOC_Os04g54930 | OsPGP16 |  |  |
|  | OsABCB18 | LOC_Os05g47490 | OsPGP18 |  |  |
|  | OsABCB19 | LOC_Os05g47500 | OsPGP19 |  |  |
|  | OsABCB20 | LOC_Os08g05690 | OsPGP20 |  |  |
|  | OsABCB21 | LOC_Os08g05710 | OsPGP21 |  |  |
|  | OsABCB22 | LOC_Os08g45030 | OsPGP22 |  |  |
| Maize | ZmLAX1 | GRMZM2G149481 |  |  |  |
|  | ZmLAX2 | GRMZM2G129413 |  |  |  |
|  | ZmLAX3 | GRMZM2G127949 |  |  |  |
|  | ZmLAX4 | GRMZM2G045057 |  |  |  |
|  | ZmLAX5 | GRMZM2G067022 |  |  |  |
|  | ZmABCB1 | GRMZM5G820122 |  |  |  |
|  | ZmABCB2 | GRMZM2G401769 |  |  |  |
|  | ZmABCB3 | GRMZM2G032936 |  |  |  |
|  | ZmABCB4 | GRMZM2G315375 |  |  |  |
|  | ZmABCB5 | GRMZM2G084181 |  |  |  |
|  | ZmABCB6 | GRMZM2G072850 |  |  |  |
|  | ZmABCB7 | GRMZM2G032218 |  |  |  |
|  | ZmABCB8 | GRMZM2G388539 |  |  |  |
|  | ZmABCB9 | GRMZM2G365957 |  |  |  |
|  | ZmABCB10 | GRMZM2G167658 |  |  |  |
|  | ZmABCB11 | GRMZM2G119894 |  |  |  |
|  | ZmABCB12 | GRMZM2G049351 |  |  |  |
|  | ZmABCB13 | GRMZM2G025860 |  |  |  |
|  | ZmABCB14 | GRMZM2G086730 |  |  |  |
|  | ZmABCB15 | GRMZM2G441722 |  |  |  |
|  | ZmABCB16 | GRMZM2G004748 |  |  |  |
|  | ZmABCB17 | GRMZM2G146034 |  |  |  |
|  | ZmABCB18 | GRMZM2G072071 |  |  |  |
|  | ZmABCB19 | GRMZM5G843192 |  |  |  |
|  | ZmABCB20 | GRMZM5G832772 |  |  |  |
|  | ZmABCB21 | GRMZM2G142870 |  |  |  |
|  | ZmABCB22 | GRMZM2G082385 |  |  |  |
|  | ZmABCB24 | GRMZM5G843537 |  |  |  |
|  | ZmABCB25 | GRMZM2G014089 |  |  |  |
|  | ZmABCB26 | GRMZM5G874756 |  |  |  |
|  | ZmABCB27 | GRMZM2G081573 |  |  |  |
|  | ZmABCB28 | GRMZM2G111903 |  |  |  |
|  | ZmABCB29 | GRMZM2G113203 |  |  |  |
|  | ZmABCB30 | GRMZM5G891159 |  |  |  |
|  | ZmABCB31 | GRMZM2G361256 |  |  |  |
|  | ZmABCB32 | GRMZM2G333183 |  |  |  |
|  | ZmABCB33 | GRMZM2G111462 |  |  |  |
|  | ZmABCB34 | GRMZM2G413774 |  |  |  |
|  | ZmABCB35 | GRMZM2G085236 |  |  |  |
| Sorghum | SbLAX1 | Sb01g026240 |  |  |  |
|  | SbLAX2 | Sb01g041270 |  |  |  |
|  | SbLAX3 | Sb03g040320 |  |  |  |
|  | SbLAX4 | Sb05g004250 |  |  |  |
|  | SbLAX5 | Sb09g021990 |  |  |  |
|  | SbPGP1 | Sb01g039110 |  |  |  |
|  | SbPGP2 | Sb02g019540 |  |  |  |
|  | SbPGP3 | Sb03g011860 |  |  |  |
|  | SbPGP4 | Sb03g023740 |  |  |  |
|  | SbPGP5 | Sb03g031990 |  |  |  |
|  | SbPGP6 | Sb03g032000 |  |  |  |
|  | SbPGP7 | Sb03g032030 |  |  |  |
|  | SbPGP8 | Sb03g033290 |  |  |  |
|  | SbPGP9 | Sb03g047490 |  |  |  |
|  | SbPGP10 | Sb04g006087 |  |  |  |
|  | SbPGP11 | Sb04g006090 |  |  |  |
|  | SbPGP12 | Sb04g006100 |  |  |  |
|  | SbPGP13 | Sb04g022480 |  |  |  |
|  | SbPGP14 | Sb04g031170 |  |  |  |
|  | SbPGP15 | Sb06g001440 |  |  |  |
|  | SbPGP16 | Sb06g018860 |  |  |  |
|  | SbPGP17 | Sb06g020350 |  |  |  |
|  | SbPGP18 | Sb06g030350 |  |  |  |
|  | SbPGP19 | Sb07g003510 |  |  |  |
|  | SbPGP20 | Sb07g003520 |  |  |  |
|  | SbPGP21 | Sb07g023730 |  |  |  |
|  | SbPGP22 | Sb09g002940 |  |  |  |
|  | SbPGP23 | Sb09g027320 |  |  |  |
|  | SbPGP24 | Sb09g027330 |  |  |  |
