# Supplementary material for: Comprehensive Analysis and Expression Profiling of the OsLAX and OsABCB Auxin Transporter Gene Families in Rice (Oryza sativa) under Phytohormone Stimuli and Abiotic Stresses
Source: Front Plant Sci. 2016 May 3;7:593. doi: 10.3389/fpls.2016.00593 (PMC4853607; doi:10.3389/fpls.2016.00593)
Supplement: Table S2 — Primers used in qPCR analyses. [file Table2.DOCX]

| **Table S2.** Primers used in qRT-PCR in this study. | |  |
| --- | --- | --- |
|  |  |  |
| **Gene name** | **Forward primer (5’-3’)** | **Reverse primer (5’-3’)** |
| *OsLAX1* | GTGTGGGAGAAGGTGATCGG | AGTTGATGGGGCCGAAGAAG |
| *OsLAX2* | CTCATCGGACTCCACGACTG | AGTTGATGGGCCCGAAGAAG |
| *OsLAX3* | ACTACATCAACGACCGGCTG | ACCAGATGCGGTAGTTGTGG |
| *OsLAX4* | ACTGGAGGAATGTGGGACTG | AATGACGGGATGAAGACGGT |
| *OsLAX5* | TCCCGTCCTTCCACAACTAC | TAGAGCACCATCGTCTTGCT |
| *OsABCB1* | CCACAGGAATGAAGCCCGAT | TGACCGACCAATGCAACTGT |
| *OsABCB2* | TTGCGCTGCTTGAAAGGTTC | TACGAAGCCAGCTGACCTTG |
| *OsABCB3* | TTTGACGGTGTGGAGCTTGA | ACGGATGGTGTCGTTGAACA |
| *OsABCB4* | TGGATGAGGCTACAAGTGCC | TGCAATCACATCTGCCCCTT |
| *OsABCB5* | CTTTTGCGGCAGATTGGAGG | CGCAACTTGGTTTGCATCCT |
| *OsABCB6* | TCCGGCGTTTAGTTGGAGAC | TTCCAGTCCGCTATCATCGC |
| *OsABCB7* | TGCTCTCGATGCACAGTCAG | TGTGTTCAGTCGATGTGCCA |
| *OsABCB8* | GAGGCCCTCGACACATTGAT | TGAATCATGCGTTCCCTGCT |
| *OsABCB9* | GGGACAGTTGTGGAGAAGGG | GCAAGCTGTGTTGTGATGCA |
| *OsABCB10* | CTGTTGCGCTTGTTGGTGAA | AGAAAGATGGCGCCAGAGTC |
| *OsABCB11* | GGAGATGGCCAGCTTCAAGT | GAACACCGACGACACCATCT |
| *OsABCB12* | GGAGATGGCCAGCTTCAAGT | GAACACCGACGACACCATCT |
| *OsABCB13* | GGGTTTCAGGATCGGGGAAG | CTCGGCCATCCAACAGAACT |
| *OsABCB14* | TCAAGGTCTTCGTCGTGCTC | TGAGGATGGCAAAGACGGAC |
| *OsABCB15* | CGTGCCTTAGTCCCATGTGA | TCAACCGTTCCATTGCTCGA |
| *OsABCB16* | AGTCTTGGCCAGGCATTCTC | TCTGGCGAATGACCTCCAAC |
| *OsABCB17* | TGATGAACAGGACCACGGTG | TCATCCCGTTCTTCACCACC |
| *OsABCB18* | TACGGACTGTCGCTTCGTTC | ATATGCCGAAACCCAAGCCA |
| *OsABCB19* | TCTCAACAATCCAAGGCGCT | TGGCGAAGTTCCACAAGTGA |
| *OsABCB20* | TGGAGGCGAACATTCACGAA | TTCTGCCCTCCTGAAAGCTG |
| *OsABCB21* | ATTAGTTGGGCCCAGTGGTG | CGTCAACAAGTACCTGCCCT |
| *OsABCB22* | CTGTCCAGCACCTCTTCTGG | GTCCTCCATGTCGAACCAGG |
| *OsActin1* | ATCCTTGTATGCTAGCGGTCGA | ATCCAACCGGAGGATAGCATG |
