# Supplementary material for: Comprehensive Analysis and Expression Profiling of the OsLAX and OsABCB Auxin Transporter Gene Families in Rice (Oryza sativa) under Phytohormone Stimuli and Abiotic Stresses
Source: Front Plant Sci. 2016 May 3;7:593. doi: 10.3389/fpls.2016.00593 (PMC4853607; doi:10.3389/fpls.2016.00593)
Supplement: Table S3 — Percent ORF nucleotide (bottom-left) and amino acid (up-right, bold) identities among OsLAXs. [file Table3.DOCX]

| **Table S3** Percent ORF nucleotide (bottom-left) and amino acid (up-right, bold) identities of *OsLAXs*. | | | | | | | | | | | | | | | | | | | | | | | | |
| --- | --- | --- | --- | --- | --- | --- | --- | --- | --- | --- | --- | --- | --- | --- | --- | --- | --- | --- | --- | --- | --- | --- | --- | --- |
|  |  |  |  |  |  |  |  |  |  |  |  |  |  |  |  |  |  |  |  |  |  |  |  |  |
| ***OsLAX*** | *1* | *2* | *3* | *4* | *5* |  |  |  |  |  |  |  |  |  |  |  |  |  |  |  |  |  |  |  |
| *1* | *** | **76** | **88** | **72** | **73** |  |  |  |  |  |  |  |  |  |  |  |  |  |  |  |  |  |  |  |
| *2* | 78 | *** | **78** | **82** | **79** |  |  |  |  |  |  |  |  |  |  |  |  |  |  |  |  |  |  |  |
| *3* | 86 | 77 | *** | **75** | **76** |  |  |  |  |  |  |  |  |  |  |  |  |  |  |  |  |  |  |  |
| *4* | 76 | 83 | 75 | *** | **77** |  |  |  |  |  |  |  |  |  |  |  |  |  |  |  |  |  |  |  |
| 5 | 77 | 80 | 75 | 79 | *** |  |  |  |  |  |  |  |  |  |  |  |  |  |  |  |  |  |  |  |
