# Supplementary material for: Comprehensive Analysis and Expression Profiling of the OsLAX and OsABCB Auxin Transporter Gene Families in Rice (Oryza sativa) under Phytohormone Stimuli and Abiotic Stresses
Source: Front Plant Sci. 2016 May 3;7:593. doi: 10.3389/fpls.2016.00593 (PMC4853607; doi:10.3389/fpls.2016.00593)
Supplement: Table S5 — Gene information of OsLAXs and OsABCBs. [file Table5.DOCX]

**Table S5.** Gene information of *OsLAX*s and *OsABCBs*.

| **Gene Name** | **Locus ID^a^** | **Protein^b^** | | | **Chromosome^a^** | **Location^a^ (Mb)** | **Strand direction^a^** | **Transmembrane helices^c^** | **Subcelluar localization^d^** |
| --- | --- | --- | --- | --- | --- | --- | --- | --- | --- |
|  |  | **length** | **MW**(kDa) | **PI** |  |  |  |  |  |
| OsLAX1 | LOC_Os01g63770 | 492 | 54.762 | 8.048 | Chr1 | 6.27 | 1 | 10 | Plas |
| OsLAX2 | LOC_Os03g14080 | 524 | 58.083 | 9.054 | Chr3 | 28.77 | 1 | 10 | plas |
| OsLAX3 | LOC_Os05g37470 | 503 | 55.663 | 8.544 | Chr5 | 8.03 | -1 | 11 | plas |
| OsLAX4 | LOC_Os10g05690 | 547 | 59.846 | 8.56 | Chr10 | 20.35 | -1 | 10 | cyto |
| OsLAX5 | LOC_Os11g06820 | 480 | 52.957 | 9.196 | Chr11 | 25.68 | -1 | 9 | cyto/plas |
| OsABCB1 | LOC_Os01g18670 | 1,285 | 138 | 8.235 | Chr1 | 32.75 | -1 | 11 | plas |
| OsABCB2 | LOC_Os01g34970 | 843 | 92.672 | 8.486 | Chr1 | 23.92 | 1 | 7 | plas |
| OsABCB3 | LOC_Os01g35030 | 1,159 | 126.539 | 7.038 | Chr1 | 23.89 | 1 | 8 | plas |
| OsABCB4 | LOC_Os01g50080 | 1,154 | 126.193 | 7.452 | Chr1 | 14.49 | 1 | 8 | plas |
| OsABCB5 | LOC_Os01g50100 | 1,181 | 128.105 | 8.582 | Chr1 | 14.48 | -1 | 8 | plas |
| OsABCB6 | LOC_Os01g50160 | 1,274 | 137.7 | 7.087 | Chr1 | 14.44 | 1 | 11 | plas |
| OsABCB7 | LOC_Os01g52550 | 1,234 | 134.572 | 8.646 | Chr1 | 13.08 | -1 | 9 | plas |
| OsABCB8 | LOC_Os01g74470 | 1,397 | 154.213 | 6.666 | Chr1 | 0.14 | -1 | 12 | cyto |
| OsABCB9 | LOC_Os02g09720 | 1,245 | 135.053 | 8.296 | Chr2 | 30.94 | -1 | 11 | plas |
| OsABCB10 | LOC_Os02g21750 | 788 | 85.506 | 8.589 | Chr2 | 23.01 | -1 | 4 | plas |
| OsABCB11 | LOC_Os02g46680 | 1,264 | 138.927 | 9.287 | Chr2 | 7.44 | -1 | 11 | plas |
| OsABCB12 | LOC_Os03g08380 | 1,482 | 158.870 | 8.713 | Chr3 | 32.13 | 1 | 9 | chlo |
| OsABCB13 | LOC_Os03g17180 | 1,411 | 155.645 | 6.287 | Chr3 | 26.85 | -1 | 13 | plas |
| OsABCB14 | LOC_Os04g38570 | 1,259 | 136.773 | 8.308 | Chr4 | 12.58 | -1 | 10 | plas |
| OsABCB15 | LOC_Os04g40570 | 1,279 | 138.422 | 8.716 | Chr4 | 11.41 | 1 | 8 | plas |
| OsABCB16 | LOC_Os04g54930 | 649 | 69.292 | 7.187 | Chr4 | 2.83 | -1 | 6 | plas |
| OsABCB17 | LOC_Os05g04610 | 524 | 56.822 | 6.622 | Chr5 | 27.79 | 1 | 4 | chlo |
| OsABCB18 | LOC_Os05g47490 | 1,302 | 140.604 | 7.431 | Chr5 | 2.75 | 1 | 8 | plas |
| OsABCB19 | LOC_Os05g47500 | 1,213 | 131.31 | 8.173 | Chr5 | 2.74 | 1 | 8 | plas |
| OsABCB20 | LOC_Os08g05690 | 1,245 | 136.717 | 7.191 | Chr8 | 25.39 | 1 | 11 | plas |
| OsABCB21 | LOC_Os08g05710 | 1,242 | 135.66 | 5.756 | Chr8 | 25.38 | 1 | 5 | plas |
| OsABCB22 | LOC_Os08g45030 | 1,344 | 145.196 | 8.468 | Chr8 | 0.18 | 1 | 12 | cyto |

a, gene information was retrieved from the *Oryza sativa* v7.0 genome annotation (phytozome 10.3: http://phytozome.jgi.doe.gov/pz/portal.html)

b, protein profiles are calculated using the Lasergene (v7.1) software.

c, transmembrane helices were predicted using the TMHHM Server v2.0 (http://www.cbs.dtu.dk/services/TMHMM/).

d, subcellular localization was predicted by WoLF PSORT (http://www.genscript.com/psort/wolf_psort.html). Plas, plasamemebrane; cyto, cytoplasm；chlo, chloroplast.
